# Effects of Regulated Deficit Irrigation at Key Growth Stages on Yield and Water Use Efficiency of Foxtail Millet in the Loess Plateau

**DOI:** 10.3390/plants15142128

**Published:** 2026-07-10

**Authors:** Shuqing Guo, Fei Han, Jiakun Yan, Suiqi Zhang

**Affiliations:** 1 The Research Center of Soil and Water Conservation and Ecological Environment, Chinese Academy of Sciences and Ministry of Education, Yangling 712100, China; gsq055069@nwafu.edu.cn; 2 State Key Laboratory of Soil and Water Conservation and Desertification Control, Institute of Soil and Water Conservation, Chinese Academy of Sciences and Ministry of Water Resources, Yangling 712100, China; 3 University of Chinese Academy of Sciences, Beijing 100049, China; 4 College of Resources and Environment, Shandong Agricultural University, Taian 271000, China; hanfeiyanzhou@163.com; 5 Shaanxi Key Laboratory of Ecological Restoration in Shanbei Mining Area, College of Life Science, Yulin University, Yulin 719000, China; himingse@163.com; 6 State Key Laboratory of Soil and Water Conservation and Desertification Control, Northwest A&F University, Yangling 712100, China; 7 College of Natural Resources and Environment, Northwest A&F University, Yangling 712100, China

**Keywords:** foxtail millet, regulated deficit irrigation, grain yield, water use efficiency

## Abstract

Regulated deficit irrigation (RDI) is an important water-saving strategy in arid regions. To quantify the effects of RDI on foxtail millet yield and water use efficiency and determine an optimal RDI strategy, a three-year field trial was carried out over dry, normal, and wet rainfall years in the Loess Plateau. Full irrigation throughout the whole growth period served as the control, whereas mild, moderate, and severe deficit irrigation treatments were conducted at the jointing–booting stage, heading–flowering stage, and across the whole growing period, respectively. The results indicate that the effects of RDI on foxtail millet yield varied with crop growth stage and deficit severity. During the heading–flowering stage, mild RDI showed statistically similar grain yield and WUE relative to those under full irrigation. In normal and wet years, moderate and severe RDI had no statistically significant effects on grain yield and WUE. Additionally, moderate and severe RDI significantly improved irrigation water use efficiency by 19.94–28.50% and 34.35–47.72%, respectively. The primary reason is that RDI at this stage maintained root development and led to only limited suppression of plant growth. In contrast, moderate and severe RDI at the jointing–booting stage or throughout the whole growth period significantly inhibited root establishment and plant development, reduced dry matter accumulation, and consequently led to substantial yield losses. The inhibitory effect became more pronounced with increasing deficit severity. Specifically, severe RDI at the jointing–booting stage and throughout the entire growth period significantly reduced yield by 19.35–54.98% and 31.47–100%, respectively. Furthermore, to identify the optimal RDI regime adaptable to variable rainfall years, a multi-model comprehensive evaluation system based on yield and WUE was established by integrating three individual evaluation models, including the membership function method, TOPSIS, and grey relational analysis, with the Fuzzy–Borda combined evaluation model. The result showed that the heading–flowering stage is the critical period for implementing RDI in foxtail millet in the Loess Plateau. Mild RDI during this stage is preferred because it maintains stable yield and WUE while substantially reducing irrigation amount over various rainfall years. Additionally, moderate and severe RDI can also maintain stable yield while significantly improving irrigation water use efficiency in normal and wet years.

## 1. Introduction

Foxtail millet (*Setaria italica* L.) serves as a major crop supporting the green development of dryland ecological agriculture, with strong tolerance to drought and infertile soils [1]. As one of the main production zones for foxtail millet in China, the Loess Plateau remains a region where water scarcity is the limiting factor for yield improvement. Under the background of ongoing global climate change, rising temperatures and increasingly erratic precipitation during the foxtail millet growing season have become more pronounced. These changes increase crop physiological water demand while reducing available water resources, thereby threatening the stability of foxtail millet production in arid zones [2,3,4,5]. Moreover, local agricultural production is still characterized by extensive irrigation practices and low water productivity, leading to a persistent mismatch between high water input and low utilization efficiency. This mismatch not only exacerbates the contradiction between water supply and demand but also leads to negative public perceptions of agricultural water use. Consequently, during years of severe water scarcity, agricultural water allocations are often diverted to meet the demands of non-agricultural sectors [6]. Conventional irrigation regimes typically usually provide full or excessive water based on crop water requirement, which readily leads to excessive soil moisture and accelerates unproductive evaporation from the soil [7]. In addition, excessive irrigation causes nutrient leaching from farmland soils, thereby exerting negative impacts on the ecological environment [8]. Therefore, it is crucial to optimize irrigation strategies, improve agricultural water use efficiency, and reduce non-beneficial water consumption for alleviating water supply–demand imbalances in the Loess Plateau, safeguarding foxtail millet yields and farmer incomes and promoting regional sustainable agricultural development [9,10].

Regulated deficit irrigation (RDI) is an effective water-saving irrigation practice developed to address water scarcity and low water use efficiency [11]. By leveraging crop adaptive responses to mild water stress, RDI can drastically reduce irrigation inputs, while minimal yield loss [12] thereby significantly improves the water productivity. It is therefore regarded as an important strategy for conserving water resources, maintaining ecological sustainability, and mitigating water use conflicts in arid regions [13]. The core principle of RDI is to impose controlled moderate water deficit at specific crop growth stages to trigger adaptive physiological adjustments, optimize photosynthate partitioning, and minimize adverse impacts on yield [14]. Meanwhile, compensatory growth effect following rewatering further offsets yield losses caused by water deficit [5,15]. Furthermore, RDI can change the distribution of soil moisture, regulate root development and water uptake capacity, and ultimately enhance whole-plant water use efficiency [15,16]. Despite its potential of both water saving and improving water productivity, the actual promotion and application of RDI remains limited [7,17]. One of the main reasons is the lack of systematic studies on crop responses to different deficit levels under diverse climatic and soil conditions, leaving farmers without reliable RDI guidelines and thus perpetuating conventional irrigation practices [17,18,19]. Moreover, the effects of RDI are strongly stage dependent: crop sensitivity to water stress varies among growth stages, and the timing of water regulation directly affects evapotranspiration, dry matter accumulation, and allocation to grains. Consequently, insufficient water supply during key growth period often leads to a simultaneous decline in both yield and water use efficiency [20].

At present, the optimal timing and level of RDI for foxtail millet in the Loess Plateau remain unclear. Moreover, existing studies mainly focus on crop responses to deficit treatments applied at a single growth stage or under one fixed water deficit level, while systematic comparisons of varied deficit gradients applied at key growth stages, including the jointing–booting stage, the heading–flowering stage, and the entire growing period, are still lacking. Meanwhile, the quantitative relationships between varying deficit levels and foxtail millet growth traits, yield formation, and water use efficiency remain poorly understood, limiting the formulation of precise irrigation strategies for field production. Accordingly, this study used foxtail millet as the experimental material and conducted field experiments in Yulin. The main objectives are (i) to investigate the effects of different degrees of RDI applied at the jointing–booting stage, heading–flowering stage, and the whole growth period on foxtail millet growth, dry matter accumulation, grain yield, and water use efficiency and (ii) to determine the optimal RDI regime for foxtail millet under drip irrigation in the Loess Plateau by comprehensively weighing both yield and water use efficiency. The findings are expected to offer a theoretical basis for effective water management in Loess Plateau.

## 2. Results

### 2.1. Effects of Regulated Deficit Irrigation on Foxtail Millet Plant Growth

**Figure 1:**
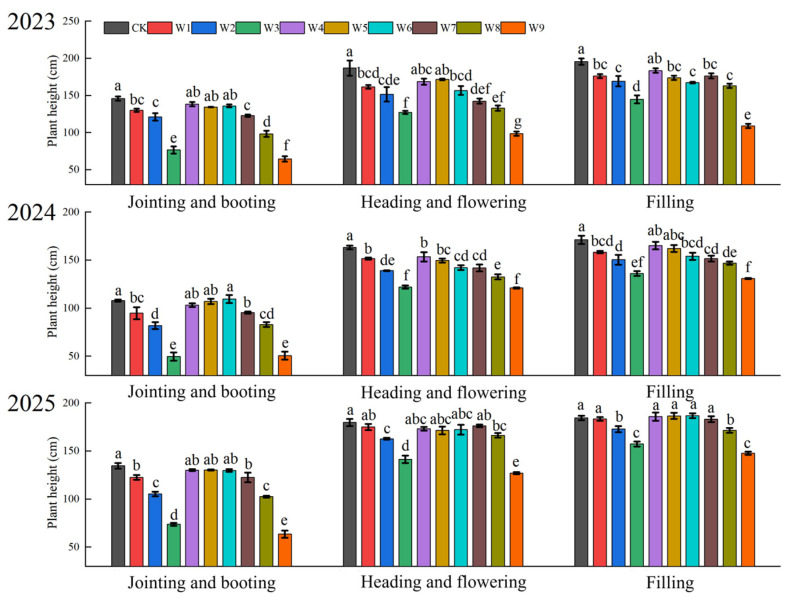
Effect of different regulated deficit irrigation on PH of foxtail millet. Different lower case letters denote significant differences among treatments at *p* < 0.05.

**Figure 2:**
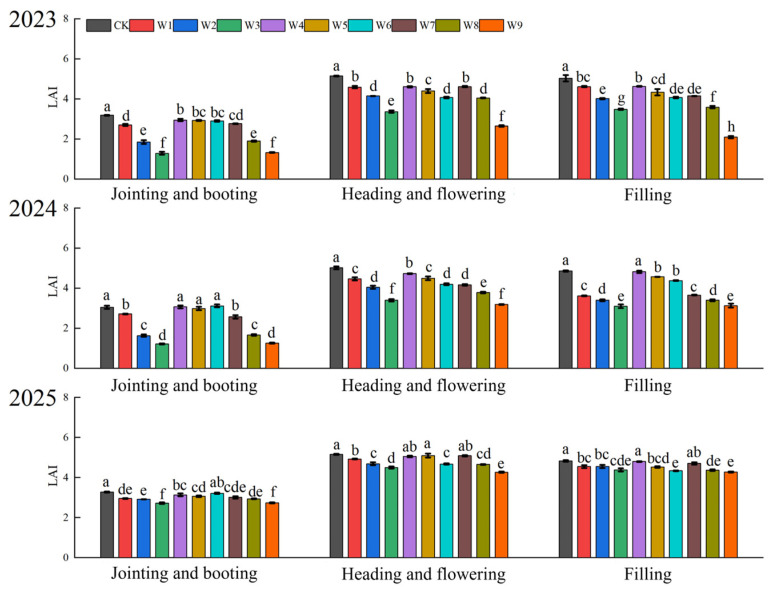
Effect of different regulated deficit irrigation on LAI of foxtail millet. Different lower case letters denote significant differences among treatments at *p* < 0.05.

The three-year experimental results showed that the foxtail millet plant height (PH) gradually increased with the growth process and stabilized at the grain-filling stage (Figure 1). The leaf area index (LAI) first increased and then declined, with the maximum value observed at the heading–flowering phase (Figure 2). Regulated deficit irrigation (RDI) applied throughout the whole growth period significantly reduced the PH and LAI, and the reduction magnitude increased with the increasing deficit severity. As crop growing season progressed, the inhibitory effects of different deficit levels on the PH and LAI gradually weakened. At the grain-filling stage, except for the mild deficit irrigation (W7) in 2025, the PH and LAI under all other RDI treatments remained significantly lower than those under the control. Among these, the severe deficit treatment (W9) caused the largest reductions, with the PH and LAI decreased by 19.86–44.29% and 11.39–58.27%, respectively, compared with the control. RDI applied at the jointing–booting stage also significantly reduced the PH and LAI, and the reduction magnitude increased with the increasing deficit severity. At the grain-filling stage, except for mild deficit treatment (W1) in 2025, the PH and LAI under all other treatments were still significantly lower than those under the control. Severe deficit treatment (W3) caused the greatest reductions, with the PH and LAI decreased by 14.67–25.98% and 9.06–36.25%, respectively, relative to the control. By contrast, RDI applied at the heading–flowering stage had little effect on the PH. In the normal rainfall year (2024), the PH under mild and moderate deficits (W4 and W5) did not differ significantly from the control; in the wet year (2025), no significant differences in the PH were detected among any RDI and the control. The LAI tended to decrease with increasing deficit severity at heading–flowering stage. In the dry year (2023), the reduction in the LAI increased as the growing season progressed, whereas in the normal and wet years, the inhibitory effect gradually decreased over time. Moreover, the LAI under mild deficit treatment (W1) recovered to a level statistically comparable to the control by the grain-filling stage.

### 2.2. Effects of Regulated Deficit Irrigation on Foxtail Millet Root Morphology

The effects of RDI on root morphology in foxtail millet varied depending on growth stage and deficit severity (Table 1). RDI applied at the jointing–booting stage or throughout the whole growth period significantly inhibited root development, as evidenced by significant reductions in the total root length, root surface area, and root diameter (*p* < 0.05). In contrast, RDI at the heading–flowering stage had no significant effect on root morphology traits. During 2023–2024, compared with the control, RDI at the jointing–booting stage significantly reduced the total root length, root surface area, and root diameter by 30.34–42.18%, 24.31–40.25%, and 8.97–20.03%, respectively. Nevertheless, in the wet year (2025), the total root length under mild deficit at the jointing–booting stage did not differ significantly different from that of the control, whereas moderate and severe deficits significantly reduced the total root length by 7.02% and 7.62%, respectively. Similarly, RDI throughout the whole growth period reduced these parameters by 7.17–41.31%, 12.72–40.05%, and 12.98–22.81%, respectively, with greater reductions observed under more severe deficit. Furthermore, moderate and severe RDI at the jointing–booting stage significantly decreased the root volume by 7.62–26.04% and 13.61–34.47%, respectively, compared with the control. Moderate and severe RDI over the whole growth period reduced the root volume by 16.56–25.49% and 29.84–37.32%, respectively.

### 2.3. Effects of Regulated Deficit Irrigation on Foxtail Millet Dry Matter Accumulation 

**Figure 3:**
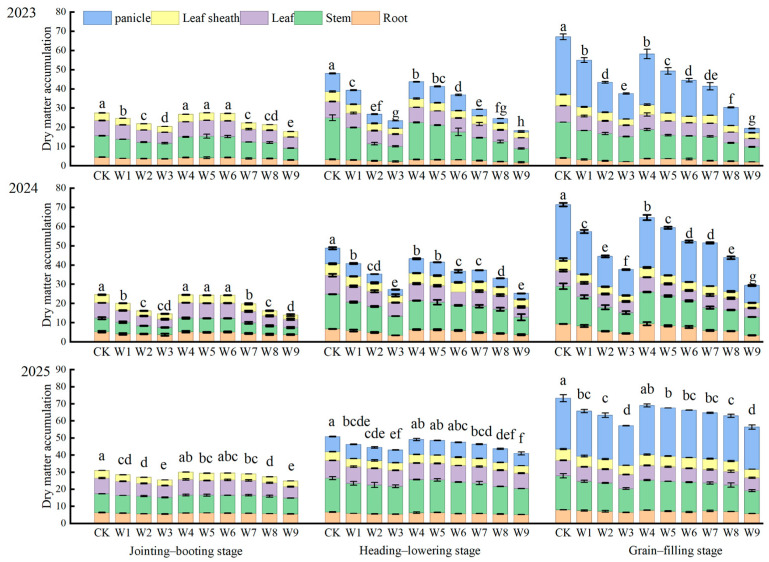
Effect of different regulated deficit irrigation treatments on dry matter accumulation of foxtail millet. Different lowercase letters indicate significant differences (*p* < 0.05).

Dry matter accumulation of foxtail millet increased continuously with the growth process and peaked at the grain-filling stage (Figure 3). Dry matter accumulation decreased with increasing severity of RDI, with stronger inhibitory effects observed under RDI applied during the whole growth period or the jointing–booting stage. During 2023–2025, compared with the control, mild, moderate, and severe RDI throughout the whole growth period significantly reduced the total dry matter by 11.60–38.41%, 13.96–54.82%, and 22.87–71.14%, respectively (*p* < 0.05). Similarly, mild, moderate, and severe RDI at the jointing–booting stage significantly reduced the total dry matter by 10.22–19.55%, 13.56–37.69%, and 21.79–47.30%, respectively. During 2023–2024, mild, moderate, and severe deficits at the heading–flowering stage significantly reduced the total dry matter by 9.34–13.34%, 16.78–26.53%, and 26.80–33.72%, respectively. In 2025, moderate and severe deficits reduced the total dry matter by 7.73% and 9.48%, respectively.

**Figure 4:**
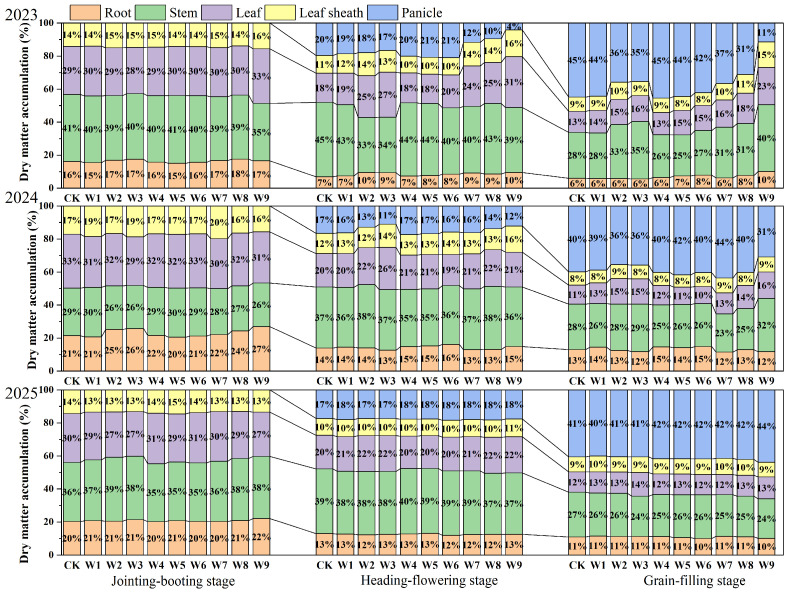
Effects of different regulated deficit irrigation treatmentss on dry weight and distribution ratio of various organs in foxtail millet. Numbers in the figure indicate the proportion of dry matter of each organ in total dry matter.

Further analysis of dry matter partitioning under different RDI treatments revealed that during 2023–2024, the proportion of dry matter allocated to panicles under severe deficit at the jointing–booting stage or throughout the whole growth period was significantly lower than that under the control (Figure 4). Under RDI at the heading–flowering stage, the proportion of panicle dry matter was also lower than that of the control, yet the difference was not statistically significant. In 2025, no significant differences in the panicle dry matter proportion were observed between any RDI compared with the control. These results indicate that the jointing–booting stage is critical for panicle differentiation in foxtail millet. Water deficit during this stage leads to poor young panicle development, which cannot be fully compensated later.

### 2.4. Effects of Regulated Deficit Irrigation on Yield and Yield Composition Characteristics

**Figure 5:**
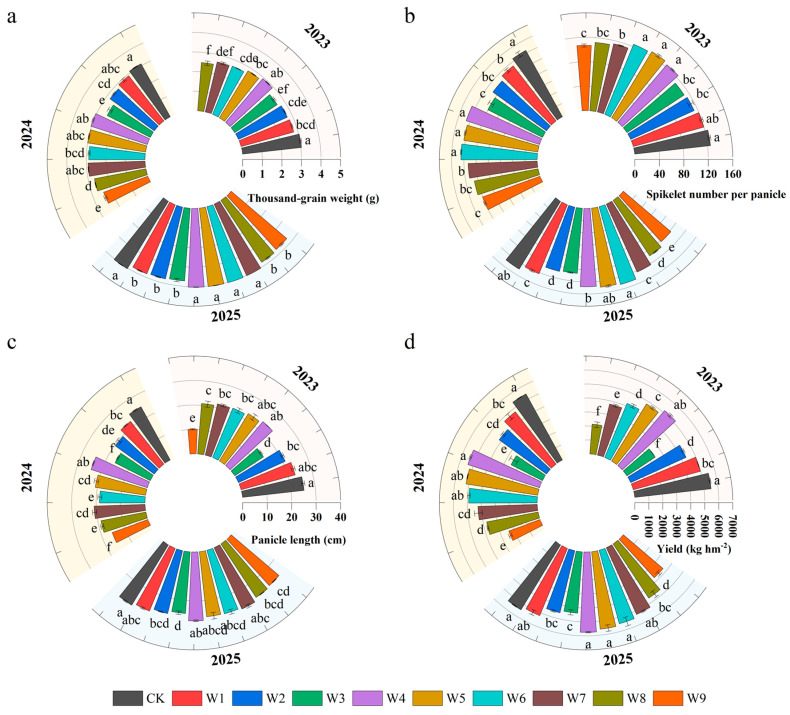
Effects of regulated deficit irrigation on grain yield and yield composition characteristics of foxtail millet. Different lowercase letters indicate significant differences (*p* < 0.05). (**a**) Thousand-grain weight; (**b**) Spikelet number per panicle; (**c**) Panicle length; (**d**) Yield.

Grain yield decreased with increasing deficit severity, with the greatest impact observed under RDI during the whole growth period, followed by RDI at the jointing–booting stage, and the least impact under RDI at the heading–flowering stage (Figure 5). Specifically, in the dry year (2023) and normal year (2024), RDI during the whole growth period significantly decreased the yield, with the severe deficit treatment (W9) reducing the yield by 55–100% compared with the control. In contrast, in the wet year (2025), mild deficit (W7) had no significant effect on the yield, whereas moderate and severe deficits (W8 and W9) reduced the yield by 14.76% and 31.47%, respectively. The direct cause of yield reduction was attributed to the deterioration of panicle traits. RDI during the whole growth period significantly reduced the panicle length (PL) and total number of spikelets (TNS), with the greatest reductions observed under W9 (11.03–60.26% for PL and 14.44–21.54% for TNS). Regarding the thousand-grain weight (TGW), W9 resulted in a complete failure of grain filling in the dry year, leading to a total crop loss. By contrast, in the normal year (2024) and wet year (2025), W7 had no significant effect on the TGW, while W8 and W9 significantly reduced the TGW by 6.35–13.28% and 7.83–22.03%, respectively.

The yield response to RDI at the jointing–booting stage exhibited a pattern similar to that of RDI throughout the whole growth period. In the dry and normal years, RDI at the jointing–booting stage significantly reduced the yield, with severe deficit (W3) causing yield reductions of 53.44–54.98%. In the wet year, mild deficit (W1) had no significant effect on the yield, whereas moderate (W2) and severe deficits (W3) reduced the yield by 14.50% and 19.35%, respectively. The yield reduction was closely associated with changes in panicle traits. Panicle length displayed a decreasing trend with increasing deficit severity at the jointing–booting stage. W2 and W3 significantly reduced the panicle length by 8.49–20.81% and 12.54–44.47%, respectively. Furthermore, W1 at the jointing–booting stage had little effect on the spikelet number per panicle, whereas W2 and W3 reduced it by 8.56–16.92% and 10.16–19.35%, respectively. In addition, RDI at the jointing–booting stage significantly reduced the TGW across all deficit levels, with the greatest reduction recorded under W3 (7.91–19.98%).

For RDI at the heading–flowering stage, in the dry year, only moderate (W5) and severe deficits (W6) significantly reduced the yield by 12.94% and 23.84%, respectively; in the normal year, only W6 slightly reduced the yield by 9.21%; and in the wet year, no significant yield reduction was observed under any deficit level. Regarding panicle traits, in the dry and normal years, the panicle length was significantly reduced under W5 and W6, with a greater reduction under W6 (13.82–25.23%). Spikelet number per panicle showed no significant change under any deficit level. The TGW was significantly reduced only under W6 in the dry and normal years (7.13–12.21%), while in the wet year, RDI at the heading–flowering stage had no significant effect on the TGW.

### 2.5. Effects of Regulated Deficit Irrigation on Soil Water Content in Different Soil Layers of Foxtail Millet

**Figure 6:**
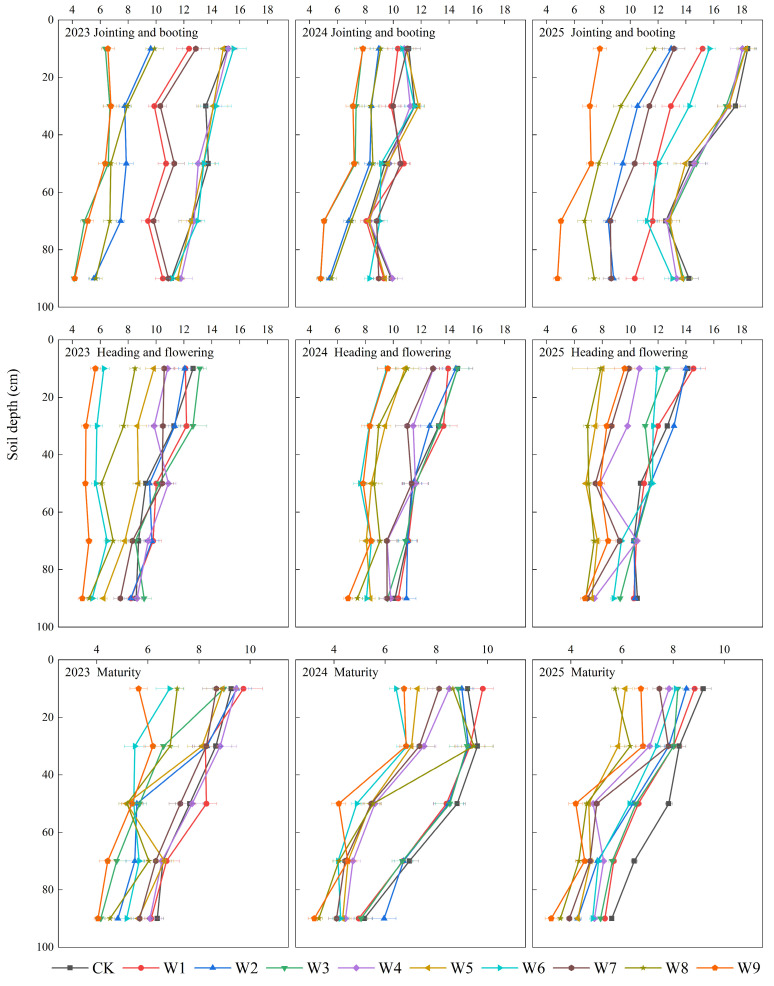
Effect of regulated deficit irrigation on soil water content within 0–100 cm soil layer during the growing period of foxtail millet.

Soil water content (SWC) generally decreased with increasing soil depth across all treatments. Under RDI, the SWC in each soil layer gradually decreased as deficit severity increased (Figure 6). At the jointing–booting stage, the SWC in each soil layer was generally higher under CK and RDI treatments at the heading–flowering stage (W4–W6), whereas RDI at the jointing–booting stage (W1–W3) and continuous RDI throughout the whole growth period (W7–W9) significantly reduced the SWC, with a clear gradient of decrease as deficit severity increased. Specifically, in the dry year, the SWC in the 0–20 cm layer at the jointing–booting stage decreased by 18.30%, 36.46%, and 57.93% under W1, W2, and W3, respectively, compared with CK; corresponding reductions under W7, W8, and W9 were 15.06%, 34.61%, and 56.74%. In the normal year, the SWC under W1, W2 and W3 decreased by 7.08%, 19.32% and 29.85%, respectively, and under W8 and W9 by 18.12% and 29.55%, respectively. In the wet year, the SWC under W1, W2 and W3 decreased by 5.96%, 17.59% and 29.78%, respectively, and reductions under W7, W8 and W9 were 14.94%, 28.72% and 36.31%, respectively.

At the heading–flowering stage, CK and W1–W3 maintained relatively high SWC following rewatering, whereas the SWC under RDI at the heading–flowering stage was significantly lower than that of CK. In the dry year, the SWC of the 0–20 cm soil layer under W4–W6 was significantly reduced by 14.38%, 22.20% and 50.55%, respectively, compared with CK. In the normal year, the SWC under W4–W6 was reduced by 11.84%, 25.87% and 34.70%, respectively, whereas in the wet year, the corresponding reductions were 10.68%, 24.61% and 43.28%, respectively. Greater reductions in SWC were observed under RDI throughout the whole growth period. In the dry year, the SWC under W7–W9 was reduced by 16.35%, 32.86% and 55.29%, respectively, compared with CK; in the normal year, by 12.05%, 24.71% and 34.22%, respectively; and in the wet year, by 15.56%, 29.92% and 44.13%, respectively. At maturity, with the cessation of irrigation and continuous water consumption by the crop, the SWC across all treatments decreased further, and severe deficit treatments at different growth stages still maintained the lowest SWC levels, indicating that the effects of water deficit persisted through the maturity stage.

### 2.6. Effects of Regulated Deficit Irrigation on Soil Water Consumption and Water Use Efficiency of Foxtail Millet

Total water consumption and water use efficiency (WUE) tended to decrease as deficit severity increased (Table 2). Across all treatments and precipitation conditions, the highest WUE was observed under mild deficit irrigation at the heading–flowering stage (W4), with no significant difference from the control. In the wet year, WUE under moderate (W5) and severe deficit (W6) at the heading–flowering stage was also not significantly different from the control (Table 2). Under RDI at the jointing–booting stage, WUE was significantly reduced due to substantial yield losses. Specifically, in the dry and normal years, WUE under mild (W1), moderate (W2), and severe deficits (W3) was significantly reduced by 6.66–12.18%, 17.15–19.66%, and 49.24–51.10%, respectively, compared with the control. In the wet year, WUE under W1 was not significantly different from the control, while WUE under W2 and W3 was significantly reduced by 12.70% and 15.18%, respectively. RDI throughout the whole growth period significantly reduced WUE. In the dry and normal years, WUE under mild (W7), moderate (W8), and severe deficit (W9) was significantly reduced by 14.88–27.51%, 25.42–57.14%, and 48.76–100%, respectively, compared with the control. In the wet year, WUE under W8 and W9 was significantly reduced by 10.01% and 20.44%, respectively. Precipitation use efficiency (PWUE) also decreased with increasing deficit severity. In the dry and normal years, all deficit levels at the jointing–booting stage and throughout the whole growth period significantly reduced PWUE. In the wet year, moderate and severe deficits at the jointing–booting stage and over the whole growth period significantly reduced PWUE. The effect of RDI at the heading–flowering stage on PWUE was relatively small: in the dry year, PWUE under W4 showed no statistical difference from the control; in the normal year, PWUE under W4 and W5 was comparable to the control; and in the wet year, no significant differences in PWUE were observed under any deficit level at the heading–flowering stage. Irrigation water use efficiency (IWUE) gradually increased with increasing deficit severity. Compared with the control, IWUE under W4 slightly higher than that of the control without reaching a significant level, while W5 and W6 significantly increased IWUE by 19.94–28.50% and 34.35–47.72%, respectively. In summary, compared with CK, RDI at the heading–flowering stage reduced total water consumption while maintaining relatively high water use efficiency and significantly increased irrigation water use efficiency.

### 2.7. Comprehensive Evaluation of Regulated Deficit Irrigation Strategies for Foxtail Millet

Three-year data of foxtail millet grain yield, total water consumption, water use efficiency, and irrigation water use efficiency were normalized using different methods. Specifically, min–max normalization was used for the membership function method, vector normalization was applied for the TOPSIS method, and optimal-value normalization was adopted for gray relational analysis. Based on the normalized datasets, the membership function method, TOPSIS, and gray relational analysis were separately used to evaluate and rank the different RDI treatments. The rankings obtained from the three individual evaluation methods were not entirely consistent (Table 3). Therefore, a Fuzzy–Borda combined evaluation model was further applied following the approach of Hu et al. (2022) [21] to integrate the results of the single evaluation models and generate comprehensive evaluation scores and rankings (Table 3). The results revealed that W6 achieved the highest comprehensive ranking, followed by CK, C4, and W5.

## 3. Discussion

### 3.1. Effects of Regulated Deficit Irrigation on the Growth and Development of Foxtail Millet

Roots serve as the primary link for crops to absorb water and nutrients from the soil [22,23]. Regulated deficit irrigation (RDI) modulates soil water availability, thereby affecting root growth and development, dry matter accumulation and distribution, and ultimately yield formation [14]. In this study, root morphological traits declined progressively with increasing deficit severity, indicating that water limitation constrained root system establishment. Moreover, the magnitude of this response varied considerably among growth stages. RDI at the heading–flowering stage did not significantly affect total root length, root surface area, root volume, and average root diameter compared with CK. This finding may be attributed to the fact that root morphology establishment is largely completed before the heading–flowering stage [24]. After the heading–flowering stage, few new roots are produced, the root radius changes little, and the root system is largely established [25]. Consequently, short-term water limitation during the heading–flowering stage had limited influence on root structural traits [4]. Consistent with this observation, similar findings have been reported in other cereal crops, where root morphological plasticity declines substantially after the completion of vegetative development [26]. The relatively stable root system may partly account for the drought tolerance of foxtail millet during reproductive growth and further supports the feasibility of implementing moderate deficit irrigation at this stage. By contrast, RDI imposed at the jointing–booting stage or throughout the whole growth period significantly restricted root development. This effect was likely associated with the fact that the jointing–booting stage is a period of rapid root growth in foxtail millet. Roots formed during this stage constitute the main root population supporting crop growth during the middle and late growth stages. Therefore, insufficient water supply not only restricts current root growth but also may exert lasting effects on crop performance by reducing the plant’s capacity to access soil water during reproductive development [25]. Furthermore, the experimental site consists of sandy soil, which is characterized by high porosity, rapid infiltration, and poor water-holding capacity. Under such conditions, even mild RDI may significantly reduce available soil moisture content in the active root zone [27]. The soil profile moisture depletion patterns observed in this experiment support this view, indicating that soil texture must be fully considered when developing deficit irrigation schemes for foxtail millet.

Root growth characteristics strongly influence aboveground growth by regulating water uptake. Plant height (PH) and leaf area index (LAI) are important morphological indicators of crop growth status, which determine canopy structure and light interception capacity [28]. In this study, the effects of RDI on the PH and LAI showed clear growth-stage specificity. Deficit irrigation at the jointing–booting stage and throughout the whole growth period significantly inhibited the PH and LAI, and the inhibitory effects increased with deficit severity. In contrast, deficit irrigation at the heading–flowering stage had relatively limited effects on the plant height and LAI. In normal and wet years, mild deficit irrigation during this stage did not significantly affect either the plant height or LAI. A likely explanation is that restricted root growth reduced the water uptake capacity, thereby restricting leaf expansion, accelerating functional leaves senescence, limiting canopy leaf area development, reducing the area available for light interception, and weakening canopy photosynthetic productivity [29]. Moreover, these stage-dependent differences were closely associated with seasonal precipitation patterns. The jointing–booting stage is a critical period for water demand and vegetative organ establishment in foxtail millet, during which the crop is highly sensitive to water deficit [30]. However, precipitation in Yulin is generally limited during June and July, and natural rainfall is insufficient to meet crop water demand during this stage. Inadequate irrigation can easily cause drought stress, directly restricting cell division and expansion and thus suppressing plant growth [31]. The heading–flowering stage is also sensitive to water; however, this period usually coincides with the local rainy season from late July to early August, when the precipitation is relatively abundant and stable across years [32,33]. During the three experimental years, rainfall in August was relatively sufficient in 2024 and 2025, which likely reduced the inhibitory impacts of deficit irrigation on plant growth. It is also noteworthy that the inhibitory effects of RDI on the PH and LAI gradually weakened as crop development progressed. This pattern is consistent with the compensatory growth mechanism proposed in RDI theory, where timely watering after moderate water deficit can induce compensatory growth to partly offset the negative effects of earlier water deficit through internal regulatory mechanisms [14,34].

### 3.2. Effects of Regulated Deficit Irrigation on Dry Matter Accumulation and Distribution of Foxtail Millet

Water deficit can inhibit crop growth and development, thereby decreasing dry matter accumulation and yield [35]. In the present study, the effects of RDI on dry matter accumulation varied depending on the growth period and deficit severity. Severe RDI at the jointing–booting stage or throughout the whole growth period caused the highest reductions in total dry matter accumulation and significantly decreased the proportion of dry matter allocated to panicles. This may be attributed to the fact that the jointing–booting stage serves as both a critical period of vegetative growth and the initial stage of reproductive development. Water deficit during this period restricted root establishment and canopy expansion, thereby reducing photosynthetic carbon assimilation and limiting the supply of assimilates available for panicle development. Hence, both total biomass production and reproductive sink formation were negatively affected [36]. In contrast, compared with full irrigation, mild RDI at the heading–flowering stage had only a slight inhibitory effect on dry matter accumulation, with non-significant reductions. Moderate and severe deficit irrigation at this stage significantly reduced dry matter accumulation by 7.79–26.53% and 9.55–33.72%, respectively. Notably, the proportion of panicle dry matter to total plant dry matter under RDI during the heading–flowering stage had no significant change. This indicates that although moderate and severe RDI during this stage reduced total dry matter accumulation, they did not alter the partitioning ratio of assimilates to panicles. Therefore, the source–sink relationship remained relatively coordinated, which was conducive to maintaining economic yield [14,37]. Overall, these results suggest that sufficient water supply at the jointing–booting stage is essential to ensure vigorous vegetative growth and to lay a solid foundation for subsequent yield formation. In contrast, moderate RDI during the heading–flowering stage, particularly mild to moderate RDI, can optimize dry matter partitioning without causing a significant reduction in biomass, especially in wet years.

### 3.3. Effects of Regulated Deficit Irrigation on Yield and Water Use Efficiency of Foxtail Millet

Grain yield is the primary indicator for evaluating the performance of RDI strategies [38]. This study showed that the effects of RDI on yield differed markedly among growth stages and deficit severity. RDI at the jointing–booting stage or throughout the whole growing period caused the greatest yield losses, and the magnitude of yield reduction increased with deficit severity. Specifically, in the dry year and normal years, severe RDI at the jointing–booting stage reduced yield by 53.44–54.98%, while continuous severe deficit throughout the whole growth period reduced yield by 55–100% relative to the control. This may be mainly attributed to the high sensitivity of foxtail millet to water during the jointing–booting stage, when water deficit causes irreversible damage to the root system, restricts vegetative growth, and directly affects the formation of reproductive organs [39,40]. Furthermore, when RDI persisted throughout the growing period, the cumulative effects of impaired root growth, restricted canopy development, and reduced assimilate production amplified the negative impact on grain yield [41,42]. Notably, the complete yield failure observed in the dry year of 2023 under the severe deficit throughout the whole growing period was likely driven by the combined effects of exceptionally low rainfall and the absence of supplemental irrigation, which depressed soil moisture content below the drought tolerance threshold of foxtail millet [43]. By contrast, mild RDI at the heading–flowering stage had no significant impact on yield across different rainfall years, and moderate RDI during this stage also maintained yields comparable to those of CK in normal and wet years. The result can be explained by the relatively limited impact of RDI at the heading–flowering stage on root morphology, canopy development, and assimilate partitioning. Although total biomass production was reduced, reproductive allocation remained relatively stable, allowing the grain yield to be maintained despite reduced irrigation inputs [2]. Moreover, RDI at the heading–flowering stage had no significant effect on the spikelet number per panicle, indicating that RDI during this period mainly regulated yield by affecting grain filling rather than panicle structure [44].

The main objective of water-saving agriculture is to achieve coordinated improvement in yield and water use efficiency (WUE) [45]. In this study, total water consumption decreased progressively with reduced irrigation amount, confirming that RDI effectively reduced agricultural water consumption. WUE exhibited a clear stage-specific response to water deficit. Compare with the control, moderate and severe RDI at the jointing–booting stage or throughout the whole growing period significantly decreased WUE. The reduction can be attributed to the fact that WUE depended on the balance between water savings and yield loss [46]. Specially, WUE declined when yield reductions exceeded the reduction in water consumption. In contrast, a substantial reduction in irrigation amount with relatively stable yield helps maintain or even slightly improve WUE, as observed under RDI at the heading–flowering stage. This trade-off was particularly evident for irrigation water use efficiency (IWUE) [47]. The results showed that IWUE increased progressively with increasing deficit severity at the heading–flowering stage, with moderate and severe RDI significantly increasing IWUE compared with CK. This pattern was not fully consistent with the response of WUE, mainly because IWUE is highly sensitive to reductions in irrigation input [2]. Thus, the substantial reduction in irrigation amount under moderate and severe RDI at the heading–flowering stage, combined with limited yield losses, resulted in a marked improvement in IWUE.

To further refine RDI recommendation for the Loess Plateau and avoid relying on a single indicator, three individual evaluation methods, including the membership function method, TOPSIS, and gray relational analysis, were integrated with the Fuzzy–Borda combined evaluation model based on yield, total water consumption, water use efficiency, and irrigation water use efficiency. The results identified mild RDI at the heading–flowering stage as the optimal strategy across different rainfall years. This treatment can simultaneously achieve water conservation and yield stability. Additionally, moderate and severe RDI at this stage can also maintain stable yield while significantly improving irrigation water use efficiency under normal and wet conditions. Nevertheless, the present conclusions are based on a single cultivar and a three-year field experiment. Future studies should include multiple genotypes and environments to further clarify how regulated deficit irrigation improves both yield and water productivity.

## 4. Materials and Methods

### 4.1. Experiment Site

The field experiments were conducted at the Yulin Experimental Station of Northwest A&F University (37°56′ N, 109°21′ E), Shaanxi Province from 2023 to 2025. The region has a temperate semi-arid continental monsoon climate. The soil texture at 0–20 cm is sandy loam, with a 1.49 g cm^−3^ average bulk density and 26.07% field capacity (FC). The 0–20 cm soil layer contained 5.97 g kg^−1^ of organic matter, 0.35 g kg^−1^ of total nitrogen, 7.76 mg kg^−1^ of ammoniacal nitrogen; 29.18 mg kg^−1^ of nitrate nitrogen, 114.2 mg kg^−1^ of available potassium, and 25.49 mg kg^−1^ of available phosphorus. Throughout the foxtail millet growth season, the total precipitation reached 249.80 mm in 2023, 42.51% of which occurred in June and July and 27.54% in August and September (Figure S1). In contrast, the total precipitation in 2024 was 400.80 mm, of which 57.91% occurring from August to September and only 20.58% from June to July. In 2025, total precipitation reached 655.00 mm, with June–July accounting for 42.47%, and August–September for 30.29%. Based on the multi-year average (1981–2013) of monthly precipitation (338.7 mm) from May to October in Yulin, this study adopted the precipitation anomaly percentage to classify annual precipitation types: an anomaly ≤−20% was defined as a dry year, an anomaly of ±15% as a normal year, and an anomaly ≥20% as a wet year [48]. Accordingly, 2023 was classified as a dry year, 2024 as a normal year, and 2025 as a wet year.

### 4.2. Experimental Design

The widely cultivated foxtail millet variety Jingu 21 was selected as the experimental material. Seeds were sown on 24 May 2023, 19 May 2024, and 18 May 2025, respectively. The final plant density after establishment was 300,000 plants ha^−1^. The whole growing period was divided into the seedling stage (late May to late June), jointing–booting stage (early July to early August), heading–flowering stage (early August to early September), grain-filling stage (early September to late September), and maturity stage (late September to early October). The harvest was carried out manually on October 11, 2023, October 15, 2024, and October 2, 2025, respectively. The field trial used a randomized complete block design with three biological replicates. Three irrigation levels were applied during the jointing–booting stage, the heading–flowering stage, and the entire growth period, resulting in a total of 10 treatments: ① full irrigation (CK): relative soil water content (RSWC) of the 0–20 cm layer maintained at 70–80% of field capacity (FC) throughout the growing period; ②–④ regulated deficit irrigation treatments at the jointing–booting stage: mild (W1, 60–70% FC), moderate (W2, 50–60% FC), and severe (W3, rainfed) during this stage; ⑤–⑦ regulated deficit irrigation treatments at the heading–flowering stage: mild (W4, 60–70% FC), moderate (W5, 50–60% FC), and severe (W6, rainfed) during this stage; and ⑧–⑩ regulated deficit irrigation treatments throughout the entire growth period (W7): mild (W7, 60–70% FC), moderate (W8, 50–60% FC), and severe (W9, rainfed) throughout the entire growing period. Each treatment had three replicates, giving a total of 30 experimental plots. Each plot measured 5.0 m × 4.0 m (20 m^2^) with a row spacing of 20 cm and was separated by 1.5 m from adjacent plots and 1.0 m from replicate blocks. Other agricultural practices were carried out in accordance with local planting practices.

A drip irrigation system was implemented in this study using 16 mm inner diameter drip irrigation belts with 0.3 m drip hole spacing and lateral spacing of 40 cm. Irrigation zones with independently controlled irrigation systems corresponded to the irrigation treatments. The volume of applied water was measured using water meters installed for each experimental plot. Regulated deficit irrigation was conducted at the jointing–booting and heading–flowering stages, when the RSWC within the 0–20 cm soil layer declined to 70%, 60%, and 50% of FC. The irrigation amount was calculated as follows [2,49]:

(1) $$M=10\;\times \;\rho_{b}\;\times \;h\;\times \;(\theta_{1}-\theta_{2})\;\times \;p$$

where *M* is the irrigation amount (mm), *ρ_b_* is the soil bulk density (1.49 g cm^−3^), *h* is the wet layer depth (20 cm), *θ*_1_ and *θ*_2_ represent the target and initial gravimetric soil water content, respectively (%FC), and *p* is the wetting coefficient (0.75).

### 4.3. Determination of Indicators

**Agronomic, dry matter accumulation, and yield traits.** Three uniformly growing plants were picked from each plot at different stages. Leaf area index (LAI) determinations were performed with an LAI-2000 plant canopy analyzer. Panicle length (PL) and plant height (PH) were measured using a ruler. Whole foxtail millet plants were sampled and then immediately separated into different components (including roots, stems, leaves, leaf sheaths, and panicles), placed into a kraft paper bag, oven-dried at 105 °C for approximately 30 min, and then dried at 80 °C to a constant weight before weighing with an electronic balance. At physiological maturity, three representative plants were selected for determining the total number of spikelets (TNS). After manual harvesting, the panicles were naturally air-dried, threshed, and weighed. Grain yield (GY) was calculated and converted to kilograms per hectare. Thousand-grain weight (TGW) was also determined.

**Root morphology.** Before harvest, three uniformly growing foxtail millet plants were randomly selected for each treatments, and root samples from the 0 to 100 cm soil layer were collected using a soil auger with a diameter of 9 cm. After removing adhering soil and debris, all clean roots were evenly spread on a root system scanner (Epson V850Pro, Seiko Epson Corp., Tokyo, Japan) for scanning. Root images were processed using WinRHIZO Pro 2009b software (Regent Instruments Inc., Québec City, QC, Canada) to derive root morphology parameters. After scanning, all roots were dried at 85 °C to a constant weight and subsequently weighed.

**Soil water content.** Soil water content (SWC, %) within the 0–100 cm soil layer was measured via the oven-drying method at the sowing, jointing–booting, heading–flowering, and maturity stages. In each plot, soil samples of three sampling points were collected at 20 cm depth intervals. After measuring fresh weight, all samples were then oven-dried at 105 °C to a constant weight and weighted. SWC was calculated as follows:

(2) $$\mathrm{SWC}=(m_{1}-m_{2})/(m_{2}-m_{0})$$

where $m_{1}$ is the weight of the aluminum box containing fresh soil (g), $m_{2}$ is the weight of the aluminum box containing dry soil (g), and $m_{0}$ is the weight of the empty oven-dried aluminum box (g).

**Soil water storage and water productivity.** Soil water storage (W) and water consumption (ET) were calculated:

(3) W = h × ρ × SWC × 10

(4) ET=W0−Wt+P+I+K−R−D

where *h* denotes soil depth (cm), *ρ* denotes soil bulk density (g cm^−3^), *W*0 and Wt refer to soil water storage within the 0–100 cm soil layer at sowing and harvest stages, respectively, *P* represents the precipitation during growing season (mm), *I* is the effective irrigation amount during the growing period (mm), *K* is the amount of capillary water rise (mm), *R* is the surface runoff volume (mm), and *D* is the deep percolation (mm). In this experiment, the field terrain was flat, and ridges and isolation strips were installed between plots, resulting in no surface runoff. In addition, the groundwater table was below 10 m, so both *K* and *R* were considered negligible. The planned wetted layer depth was 20 cm, and the irrigation amount was small, so that irrigation water did not infiltrate below the 120 cm soil layer; therefore, *D* was also neglected.

The water use efficiency (*WUE*), precipitation use efficiency (*PWUE*), and irrigation water use efficiency (*IWUE*) were calculated using the following equations [50]:

(5) WUE = GY/ET

(6) PWUE=GY/P

(7) IWUE=GY/I

### 4.4. Data Processing and Statistical Analysis

**Membership function method.** The membership function method, TOPSIS, and gray relational analysis were used to comprehensively evaluate the performance of RDI based on GY, ET, WUE, and IWUE from 2023 to 2025. The calculation formula of the membership function method is as follows [51]:

(8) $$x_{\left(u\right)}^{+}=\frac{x_{i}-x_{\min}}{x_{\max}-x_{\min}}$$

(9) $$x_{\left(u\right)}^{-}=\frac{x_{max}-x_{i}}{x_{\max}-x_{\min}}$$

where $x_{\left(u\right)}^{+}$ and $x_{\left(u\right)}^{-}$ represent membership function value of indicators positively and negatively correlated with the comprehensive value of the i-th treatment, respectively; $x_{i}$ is the measured value of the index for the i-th treatment; and $x_{\max}$ and $x_{\min}$ are maximum and minimum values of the measured index of the i-th treatment, respectively.

**TOPSIS method.** The calculation formula of TOPSIS is as follows [52]:

(10) $$A_{\mathrm{ij}}\;=\frac{X_{\mathrm{ij}}}{\;\sqrt{\sum_{i=1}^{10}x_{\mathrm{ij}}^{2}}}$$

(11) $$A^{+}=(A_{1}^{+},\;A_{2}^{+},\;A_{3}^{+},\ldots ,A_{n}^{+},)$$

(12) $$A^{-}=(A_{1}^{-},\;A_{2}^{-},\;A_{3}^{-},\ldots ,A_{n}^{-},)$$

(13) $$D^{+}=\sqrt{\sum_{i=1}^{9}{W_{j}\;\times \;(A_{\mathrm{ij}}-Z_{j}^{+})}^{2}}$$

(14) $$D^{-}=\sqrt{\sum_{i=1}^{9}{W_{j}\;\times \;(A_{\mathrm{ij}}-Z_{j}^{-})}^{2}}$$

(15) $$C=\frac{D^{-}}{\left(D^{+}+D^{-}\right)}$$

where $A_{\mathrm{ij}}$ is the normalized value of the index; $A^{+}$ and $A^{-}$ are the positive and negative ideal solutions in sequence; $D^{+}$ and $D^{-}$ refer to Euclidean distances from each evaluation object to the $A^{+}$ and $A^{-}$, respectively; $W_{j}$ represents the weight of index; and *C* is the closeness degree of each evaluation object to the optimal solution.

**Gray relational analysis method.** The formula for the calculation of the gray relational analysis method is as follows [53]:

(16) $${X^{\prime }}_{i}^{+}(k)\;=\;\frac{X_{i}(k)}{X_{0}(k)}$$

(17) $${X^{\prime }}_{i}^{-}(k)=\frac{X_{0}(k)}{X_{i}(k)}$$

(18) $$\Delta_{i}(k)\;=|1-{X^{\prime }}_{i}(k)|$$

(19) $$\xi_{i}(k)=\frac{\min_{i}\;\min_{k}\Delta_{i}(k)+\rho \times \max_{i}\;\max_{k}\Delta_{i}(k)}{\Delta_{i}(k)+\rho \;\times \;\max_{i}\;\max_{k}\Delta_{i}(k)}$$

(20) $$\gamma_{i}=\frac{1}{9}\sum_{k=1}^{9}\xi_{i}(k)$$

where $\Delta_{i}(k)$ denotes the absolute deviation of the indicator; $\min_{i}\;\min_{k}\Delta_{i}(k)$ and $\max_{i}\;\max_{k}\Delta_{i}(k)$ are the global minimum and maximum difference, respectively; ρ refers to the distinguishing coefficient, taken as 0.5, indicating equal importance; $\xi_{i}(k)$ is the gray relational coefficient; and $\gamma_{i}$ is the equally weighted gray relational grade.

**Statistical analysis.** Data were processed in Excel 2021, and graphs were generated with Origin 2025. One-way ANOVA with LSD tests was conducted in IBM SPSS Statistics 26.0 to test significant differences among different treatments. PCA was then applied to the comprehensive evaluation results of each treatment (membership function, TOPSIS, and gray relational analysis) using IBM SPSS Statistics 26.0; treatments were finally ranked by their first principal component score.

## 5. Conclusions

Based on a three-year field experiment across contrasting rainfall years, this study systematically evaluated the effects of RDI on the growth, yield, and water use efficiency of foxtail millet in the Loess Plateau. The results showed that the effects of RDI on foxtail millet growth and development were highly dependent on both growth stage and deficit severity. In normal and wet years, RDI at the heading–flowering stage had no significant effect on yield, while moderate and severe RDI significantly improved irrigation water use efficiency by 19.94–28.50% and 34.35–47.72%, respectively. In contrast, moderate and severe RDI at the jointing–booting stage or throughout the whole growth period significantly inhibited root morphology and plant growth, resulting in reduced biomass accumulation and substantial yield losses; these inhibitory effects became more pronounced with increasing deficit severity. The multi-model comprehensive evaluation system, integrating the membership function method, TOPSIS, gray relational analysis, and the Fuzzy–Borda combined evaluation model, indicated that mild RDI at the heading–flowering stage is the preferred water-saving strategy across various rainfall years. This approach maintains stable yield and WUE while significantly reducing irrigation amount, thus synergistically optimizing yield and water conservation. In normal and wet years, moderate and severe RDI at the heading–flowering stage achieved comparable outcomes. Future studies should include multiple cultivars, additional ecological regions, and more detailed physiological measurements to further verify the general applicability and mechanistic basis of the RDI strategy.

## Figures and Tables

**Table 1:** Effects of regulated deficit irrigation on the root morphology of foxtail millet from 2023 to 2025.

Years		Treatments	Total Root Length (cm)	Root Surface Area (cm^2^)	Root Diameter (mm)	Root Volume (cm^3^)
2023		CK	3742.61 ± 34.59 ^a^	507.72 ± 40.66 ^a^	7.44 ± 0.06 ^a^	7.26 ± 0.20 ^a^
2023		W1	2607.09 ± 80.52 ^b^	380.35 ± 1.30 ^b^	6.77 ± 0.08 ^bc^	6.40 ± 0.19 ^b^
2023		W2	2163.88 ± 57.27 ^d^	371.45 ± 5.07 ^bc^	6.46 ± 0.12 ^cd^	5.37 ± 0.19 ^cd^
2023		W3	2306.94 ± 52.23 ^cd^	335.29 ± 0.82 ^bc^	5.95 ± 0.17 ^de^	4.76 ± 0.37 ^cd^
2023		W4	3707.17 ± 30.56 ^a^	489.35 ± 7.80 ^a^	7.26 ± 0.11 ^ab^	7.14 ± 0.08 ^ab^
2023		W5	3657.27 ± 10.42 ^a^	490.36 ± 4.39 ^a^	7.25 ± 0.07 ^ab^	7.14 ± 0.11 ^ab^
2023		W6	3705.55 ± 58.87 ^a^	470.23 ± 28.48 ^a^	7.54 ± 0.07 ^a^	7.26 ± 0.33 ^a^
2023		W7	2634.70 ± 21.34 ^b^	374.69 ± 14.34 ^bc^	6.36 ± 0.16 ^cd^	6.34 ± 0.14 ^b^
2023		W8	2373.50 ± 27.29 ^c^	350.85 ± 12.81 ^bc^	6.14 ± 0.10 ^de^	5.41 ± 0.31 ^c^
2023		W9	2259.58 ± 73.57 ^cd^	318.10 ± 6.05 ^c^	5.74 ± 0.35 ^e^	4.55 ± 0.27 ^d^
2024		CK	3864.36 ± 33.32 ^a^	500.46 ± 19.85 ^a^	7.05 ± 0.57 ^a^	6.48 ± 0.34 ^a^
2024		W1	2632.04 ± 65.48 ^b^	378.82 ± 26.44 ^de^	6.03 ± 0.07 ^b^	5.64 ± 0.37 ^abc^
2024		W2	2585.87 ± 89.41 ^b^	343.49 ± 10.75 ^ef^	6.02 ± 0.09 ^b^	5.27 ± 0.39 ^cd^
2024		W3	2306.32 ± 104.97 ^c^	299.01 ± 25.71 ^f^	5.81 ± 0.21 ^b^	4.62 ± 0.25 ^d^
2024		W4	3773.85 ± 16.15 ^a^	457.46 ± 5.32 ^abc^	7.10 ± 0.07 ^a^	6.28 ± 0.22 ^ab^
2024		W5	3726.40 ± 28.25 ^a^	464.66 ± 2.24 ^ab^	7.09 ± 0.05 ^a^	6.35 ± 0.20 ^ab^
2024		W6	3850.15 ± 52.81 ^a^	419.60 ± 11.05 ^bcd^	7.49 ± 0.13 ^a^	6.11 ± 0.15 ^abc^
2024		W7	2616.57 ± 46.92 ^b^	404.13 ± 16.76 ^cd^	6.13 ± 0.03 ^b^	5.51 ± 0.27 ^bcd^
2024		W8	2544.46 ± 15.58 ^b^	364.97 ± 15.42 ^de^	6.06 ± 0.04 ^b^	5.41 ± 0.29 ^bcd^
2024		W9	2267.93 ± 107.84 ^c^	300.01 ± 9.67 ^f^	6.01 ± 0.15 ^b^	4.63 ± 0.21 ^d^
2025		CK	3961.22 ± 37.55 ^a^	452.31 ± 0.00 ^a^	6.93 ± 0.00 ^a^	7.26 ± 0.26 ^a^
2025		W1	3810.97 ± 19.56 ^ab^	367.52 ± 0.00 ^c^	5.80 ± 0.00 ^b^	7.12 ± 0.41 ^ab^
2025		W2	3683.08 ± 46.73 ^bcd^	357.73 ± 0.82 ^cd^	5.68 ± 0.00 ^bc^	6.71 ± 0.03 ^abc^
2025		W3	3659.31 ± 41.95 ^bcd^	316.73 ± 0.00 ^d^	5.28 ± 0.00 ^c^	6.27 ± 0.01 ^cd^
2025		W4	3794.16 ± 16.23 ^ab^	438.94 ± 20.92 ^ab^	6.43 ± 0.38 ^a^	6.82 ± 0.11 ^ab^
2025		W5	3794.00 ± 27.89 ^ab^	438.95 ± 24.10 ^ab^	6.65 ± 0.11 ^a^	6.81 ± 0.08 ^abc^
2025		W6	3718.30 ± 9.43 ^bc^	434.47 ± 24.99 ^ab^	6.64 ± 0.25 ^a^	6.85 ± 0.07 ^abc^
2025		W7	3677.25 ± 86.03 ^bcd^	394.76 ± 0.00 ^bc^	5.82 ± 0.00 ^b^	6.61 ± 0.14 ^bc^
2025		W8	3554.36 ± 107.78 ^cd^	387.73 ± 4.98 ^c^	5.64 ± 0.00 ^bc^	5.71 ± 0.03 ^de^
2025		W9	3508.05 ± 13.89 ^d^	365.44 ± 0.82 ^c^	5.87 ± 0.00 ^b^	5.31 ± 0.24 ^e^
**Traits**	**Variation Source**	**Mean Square**	**df**	***F*** **-Value**	***p*** **-Value**	**Estimates of Effect Size**
Total root length (cm)	Year	5,697,814.394	2	1222.459	<0.001 ***	0.976
	Treatments	2,171,599.995	9	465.914	<0.001 ***	0.986
	Year × Treatment	372,052.376	18	79.823	<0.001 ***	0.960
Root surface area (cm^2^)	Year	2040.633	2	5.555	0.006 **	0.156
	Treatments	33,093.314	9	90.087	<0.001 ***	0.931
	Year × Treatment	1495.991	18	4.072	<0.001 ***	0.550
Root diameter (mm)	Year	2.953	2	66.647	<0.001 ***	0.960
	Treatments	3.098	9	69.929	<0.001 ***	0.913
	Year × Treatment	0.111	18	2.500	0.004 **	0.429
Root volume (cm^3^)	Year	6.357	2	79.096	<0.001 ***	0.725
	Treatments	5.003	9	62.251	<0.001 ***	0.903
	Year × Treatment	0.446	18	5.544	<0.001 ***	0.625

All data are presented as the mean ± standard deviation, and different lowercase letters indicate significant differences (*p* < 0.05). ** and *** indicate significance at *p* < 0.01 and *p* < 0.001, respectively.

**Table 2:** Effects of regulated deficit irrigation during key growth stages on water use efficiency of foxtail millet from 2023 to 2025.

Years		Treatments	ET	WUE	PWUE	IWUE
2023		CK	557.18 ± 6.02 ^a^	9.86 ± 0.11 ^a^	22.00 ± 0.20 ^a^	41.64 ± 0.38 ^de^
2023		W1	540.89 ± 5.80 ^ab^	9.21 ± 0.07 ^bc^	19.94 ± 0.07 ^bc^	44.46 ± 0.15 ^cd^
2023		W2	532.02 ± 5.04 ^bc^	8.17 ± 0.21 ^d^	17.44 ± 0.35 ^d^	47.34 ± 0.96 ^bc^
2023		W3	512.98 ± 4.55 ^de^	4.82 ± 0.06 ^f^	9.91 ± 0.04 ^f^	37.49 ± 0.15 ^e^
2023		W4	537.55 ± 5.39 ^bc^	9.60 ± 0.18 ^ab^	20.67 ± 0.45 ^ab^	46.10 ± 1.00 ^cd^
2023		W5	535.40 ± 4.68 ^bc^	8.94 ± 0.16 ^c^	19.15 ± 0.40 ^c^	52.01 ± 1.08 ^b^
2023		W6	522.81 ± 4.15 ^cde^	8.00 ± 0.02 ^d^	16.76 ± 0.54 ^d^	63.42 ± 2.06 ^a^
2023		W7	525.07 ± 5.51 ^bcd^	7.15 ± 0.14 ^e^	15.03 ± 0.35 ^e^	40.81 ± 0.96 ^de^
2023		W8	505.22 ± 5.00 ^e^	4.23 ± 0.37 ^f^	8.55 ± 0.78 ^f^	41.09 ± 3.73 ^de^
2023		W9	469.29 ± 3.40 ^f^	0.00 ± 0.00 ^g^	0.00 ± 0.00 ^g^	—
2024		CK	530.47 ± 5.02 ^ab^	10.55 ± 0.06 ^a^	13.97 ± 0.19 ^a^	42.41 ± 0.56 ^de^
2024		W1	512.16 ± 6.12 ^cd^	9.27 ± 0.51 ^bc^	11.84 ± 0.60 ^cd^	42.36 ± 2.15 ^de^
2024		W2	492.11 ± 5.04 ^ef^	8.48 ± 0.16 ^cd^	10.41 ± 0.31 ^e^	45.36 ± 1.33 ^cd^
2024		W3	469.44 ± 5.84 ^g^	5.36 ± 0.64 ^e^	6.27 ± 0.73 ^f^	38.08 ± 4.43 ^e^
2024		W4	537.81 ± 4.52 ^a^	10.02 ± 0.19 ^ab^	13.44 ± 0.16 ^ab^	48.10 ± 0.59 ^cd^
2024		W5	522.63 ± 4.33 ^abc^	9.93 ± 0.05 ^ab^	12.95 ± 0.06 ^abc^	56.40 ± 0.27 ^b^
2024		W6	503.06 ± 3.89 ^de^	9.74 ± 0.21 ^ab^	12.23 ± 0.22 ^bcd^	74.26 ± 1.36 ^a^
2024		W7	520.47 ± 4.72 ^bc^	8.98 ± 0.08 ^bc^	11.66 ± 0.12 ^d^	50.82 ± 0.51 ^bc^
2024		W8	475.73 ± 4.28 ^fg^	7.87 ± 0.16 ^d^	9.34 ± 0.13 ^e^	71.99 ± 1.03 ^a^
2024		W9	440.71 ± 3.12 ^h^	5.41 ± 0.25 ^e^	5.94 ± 0.27 ^f^	—
2025		CK	989.37 ± 3.00 ^a^	6.81 ± 0.07 ^a^	10.29 ± 0.09 ^a^	51.07 ± 0.45 ^g^
2025		W1	972.82 ± 4.20 ^bc^	6.38 ± 0.19 ^abc^	9.47 ± 0.28 ^ab^	55.40 ± 1.61 ^fg^
2025		W2	950.17 ± 3.10 ^e^	5.95 ± 0.08 ^bcd^	8.63 ± 0.11 ^bc^	61.43 ± 0.77 ^ef^
2025		W3	932.39 ± 3.35 ^f^	5.78 ± 0.21 ^cd^	8.23 ± 0.28 ^c^	81.64 ± 2.81 ^c^
2025		W4	979.87 ± 3.71 ^ab^	6.88 ± 0.06 ^a^	10.29 ± 0.07 ^a^	60.16 ± 0.42 ^efg^
2025		W5	958.96 ± 3.52 ^de^	6.85 ± 0.23 ^a^	10.03 ± 0.35 ^a^	71.43 ± 2.53 ^d^
2025		W6	930.44 ± 3.25 ^fg^	6.93 ± 0.32 ^a^	9.84 ± 0.49 ^a^	97.68 ± 4.82 ^b^
2025		W7	963.84 ± 2.97 ^cd^	6.48 ± 0.07 ^ab^	9.53 ± 0.10 ^ab^	67.85 ± 0.69 ^de^
2025		W8	919.54 ± 2.87 ^g^	6.13 ± 0.27 ^bc^	8.61 ± 0.36 ^bc^	108.42 ± 4.55 ^a^
2025		W9	872.28 ± 3.18 ^h^	5.42 ± 0.15 ^d^	7.22 ± 0.21 ^d^	—
**Traits**	**Variation Source**	**Mean Square**	**df**	***F*** **-Value**	***p*** **-Value**	**Estimates of Effect Size**
ET	Year	1,894,367.066	2	64,307.893	<0.001 ***	1.000
	Treatments	7656.135	9	259.902	<0.001 ***	0.975
	Year × Treatment	271.555	18	9.218	<0.001 ***	0.734
WUE	Year	38.413	2	496.787	<0.001 ***	0.943
	Treatments	28.098	9	363.382	<0.001 ***	0.982
	Year × Treatment	6.061	18	78.383	<0.001 ***	0.959
PWUE	Year	262.507	2	1501.727	<0.001 ***	0.980
	Treatments	109.395	9	625.817	<0.001 ***	0.989
	Year × Treatment	29.518	18	168.865	<0.001 ***	0.981
IWUE	Year	5297.820	2	831.919	<0.001 ***	0.969
	Treatments	1221.369	9	191.792	<0.001 ***	0.966
	Year × Treatment	307.359	18	48.265	<0.001 ***	0.935

All data are presented as the mean ± standard deviation, and different lowercase letters indicate significant differences (*p* < 0.05). *** indicates significance at *p* < 0.001.

**Table 3:** Comparative analysis of different evaluation methods.

Treatments	Membership Functions		TOPSIS		Gray Relevance Analysis		Fuzzy Borda Combination Evaluation Model	
	Evaluation Values	Rank	Evaluation Values	Rank	Correlation Degree	Rank	Evaluation Values	Rank
CK	0.6292	4	0.4012	10	0.8720	2	27.92	2
W1	0.5932	6	0.4405	8	0.7940	5	11.26	8
W2	0.5524	8	0.4997	5	0.7339	7	7.46	10
W3	0.4250	9	0.5393	3	0.6361	9	13.04	7
W4	0.6672	3	0.4338	9	0.8672	3	24.94	3
W5	0.6963	2	0.4851	6	0.8497	4	24.69	4
W6	0.7717	1	0.6026	2	0.8756	1	42.20	1
W7	0.5743	7	0.4493	7	0.7510	6	7.68	9
W8	0.5934	5	0.6269	1	0.7296	8	24.58	5
W9	0.2508	10	0.5180	4	0.5117	10	15.52	6

## Data Availability

The original contributions presented in this study are included in the article/Supplementary Material. Further inquiries can be directed to the corresponding author.

## Supplementary Materials

The following supporting information can be downloaded at: https://www.mdpi.com/article/10.3390/plants15142128/s1.
