# Supplementary material for: Effects of Regulated Deficit Irrigation at Key Growth Stages on Yield and Water Use Efficiency of Foxtail Millet in the Loess Plateau
Source: Plants (Basel). 2026 Jul 10;15(14):2128. doi: 10.3390/plants15142128 (PMC13414876; doi:10.3390/plants15142128)
Supplement: Supplementary file 1 [file plants-15-02128-s001.zip › plants-4411144-supplementary.pdf]

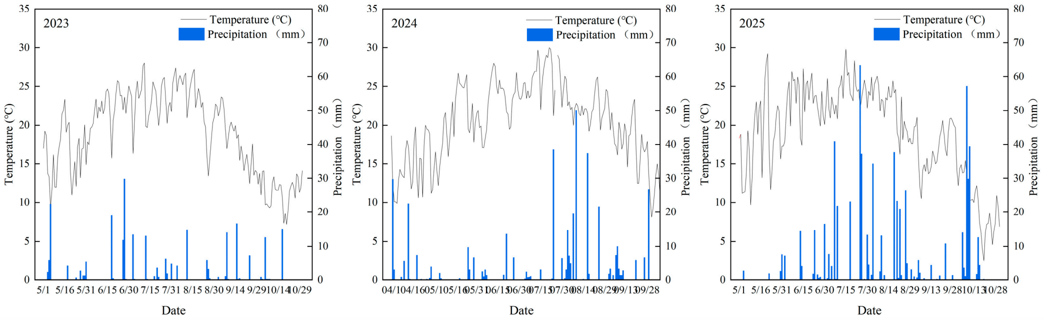

Figure S1. Dynamic changes of daily temperature (line) and precipitation (bar) throughout the foxtail millet growth season in Yulin from 2023 to 2025.
